# Development of the ECHOES national dataset: a resource for monitoring post-acute and long-term COVID-19 health outcomes in England

**DOI:** 10.3389/fpubh.2025.1513508

**Published:** 2025-03-12

**Authors:** Hester Allen, Katie Hassell, Christopher Rawlinson, Owen Pullen, Colin Campbell, Annika M. Jödicke, Martí Català, Albert Prats-Uribe, Gavin Dabrera, Daniel Prieto-Alhambra, Ines Campos-Matos

**Affiliations:** ^1^Immunisation and Vaccine Preventable Diseases Division, UK Health Security Agency, London, United Kingdom; ^2^Health Data Sciences, Centre for Statistics in Medicine, Nuffield Department of Orthopaedics, Rheumatology and Musculoskeletal Sciences (NDORMS), University of Oxford, Oxford, United Kingdom; ^3^Department of Medical Informatics, Erasmus Medical Centre University, Rotterdam, Netherlands

**Keywords:** COVID-19, post-acute COVID-19, electronic health data, data linkage, health outcomes

## Abstract

**Introduction:**

Electronic health records can be used to understand the diverse presentation of post-acute and long-term health outcomes following COVID-19 infection. In England, the UK Health Security Agency, in collaboration with the University of Oxford, has created the Evaluation of post-acute COVID-19 Health Outcomes (ECHOES) dataset to monitor how an initial SARS-CoV-2 infection episode is associated with changes in the risk of health outcomes that are recorded in routinely collected health data.

**Methods:**

The ECHOES dataset is a national-level dataset combining national-level surveillance, administrative, and healthcare data. Entity resolution and data linkage methods are used to create a cohort of individuals who have tested positive and negative for SARS-CoV-2 in England throughout the COVID-19 pandemic, alongside information on a range of health outcomes, including diagnosed clinical conditions, mortality, and risk factor information.

**Results:**

The dataset contains comprehensive COVID-19 testing data and demographic, socio-economic, and health-related information for 44 million individuals who tested for SARS-CoV-2 between March 2020 and April 2022, representing 15,720,286 individuals who tested positive and 42,351,016 individuals who tested negative.

**Discussion:**

With the application of epidemiological and statistical methods, this dataset allows a range of clinical outcomes to be investigated, including pre-specified health conditions and mortality. Furthermore, understanding potential determinants of health outcomes can be gained, including pre-existing health conditions, acute disease characteristics, SARS-CoV-2 vaccination status, and genomic variants.

## 1 Introduction

SARS-CoV-2 infection typically causes acute respiratory symptoms, and health outcomes associated with the acute period following symptom onset are well understood and described in the scientific literature (1, 2).

Increasingly, data-driven approaches are being used to characterise and determine the prevalence, symptoms, and risk factors of long-term COVID-19 health outcomes (3–5). Electronic health records can be used to understand the diverse presentation of post-acute COVID-19 in representative populations captured through routine care (1, 6, 7).

In England, during the COVID-19 pandemic, robust, national surveillance systems were developed to collect comprehensive data to monitor trends in COVID-19, including SARS-CoV-2 testing, cases, and test positivity (8). Using these COVID-19 surveillance data alongside other routinely collected surveillance data, the UK Health Security Agency (UKHSA), in collaboration with the University of Oxford, has developed the Evaluation of Post-acute COVID-19 Health Outcomes (ECHOES) dataset.

The purpose of the ECHOES dataset is to monitor how an initial SARS-CoV-2 infection episode is associated with changes in the risk of a range of health outcomes, including those recorded through secondary healthcare services and death in the period following the acute phase of COVID-19 disease.

ECHOES is a dataset designed as a longitudinal cohort comprising data for individuals tested for SARS-CoV-2 in England throughout the COVID-19 pandemic, with information on health outcomes following their COVID-19 test. Information on health outcomes will be updated periodically, on a quarterly basis. Therefore, the ECHOES dataset can identify post-acute health outcomes among those with COVID-19 disease at different time intervals following their initial infection, including long-term health outcomes of COVID-19 occurring beyond the end of the pandemic period.

This dataset benefits from the use of a range of national-level data, including comprehensive SARS-CoV-2 testing data, individuals testing positive and negative in both community and hospital settings, genomic sequencing data, and vaccination records, alongside extensive demographic, socio-economic, and health-related information.

The use of routine health datasets allows health outcomes to be assessed using a range of statistical and epidemiological methods. These can be used to compare the risk outcomes between persons with SARS-CoV-2 infection and test-negative controls, taking into account outcome occurrence in the exposure and follow-up periods.

As part of the remit to diagnose, control, prevent, and describe trends in communicable diseases and other risks to public health, UKHSA processes personal information to develop and maintain surveillance systems and conduct infection control activities in accordance with Regulation 3 SI 1438 Control of Healthcare Information Regulations 2002 (s251), as the legal basis for data processing in the absence of individual consent (9, 10).

## 2 Methods

The ECHOES dataset is a longitudinal cohort capturing individuals who tested for SARS-CoV-2 between March 2020 and April 2022. This cohort is linked to multiple data sources to obtain information on health outcomes occurring in the post-acute phase (defined as >28 days after the test) and factors associated with COVID-19 infection and health outcomes. The dataset is built in two main steps: cohort building and linkage to additional data sources. Data processing is carried out using SQL, and statistical analysis is carried out using R.

All data are processed within secure UKHSA servers. Whilst person-identifying information (PII) is required in data processing to identify unique records within data sources, across multiple data sources, and to link records from different data sources, following data linkage, all PII are removed from the dataset.

### 2.1 Data sources

The data sources utilised to build the ECHOES dataset are all routinely collected national-level surveillance, administrative, and healthcare data for which UKHSA are the owners or processors. These include COVID-19 testing data, the National Immunisation Management System (NIMS), the Office of National Statistics Death Registration data, and Hospital Episode statistics.

COVID-19 testing data are collected through two data systems—the Second-Generation Surveillance System (SGSS) and the Unified Sample Dataset (USD).

Second generation surveillance system (SGSS) collects infectious disease laboratory test information from diagnostic laboratories in England (11, 12). SARS-CoV-2 testing data in SGSS includes polymerase chain reaction (PCR) tests from NHS settings (including NHS patients and staff) and PCR and lateral flow device (LFD) tests from community settings, submitted by individuals via an NHS website.

SGSS records are validated and enhanced using the NHS spine, a database containing PII for everyone who has ever had contact with the NHS (13). Positive test records are converted to cases and infection episodes, whereby COVID-19 episodes are defined as 91 days apart (11).

Where available, SARS-CoV-2 genomic information is included in SGSS (14). In England, during the pandemic, genomic investigation (whole genome sequencing, reflex assays, and S-gene target investigation) and variant assignment of a sample of eligible SARS-CoV-2 PCR samples were coordinated by the COVID-19 Genomics UK consortium (COG UK).

The Unified sample dataset (USD) is a repository for positive and negative COVID-19 testing data in England, including all PCR and LFD tests (8, 15). Test records are enriched with patient information from the NHS spine (13).

The National Immunisation Management System (NIMS) was developed in 2020 as part of the national influenza and COVID-19 vaccination programme. It collects information on individuals eligible for SARS-CoV-2 vaccination and administered vaccinations in England (16, 17).

NIMS contains both person records for all individuals issued with an NHS number in England (the NIMS population denominator), who were eligible for vaccination when the vaccination programme began in December 2020, therefore acting as a national COVID-19 vaccination register, and detailed vaccination records for all individuals who have received at least one dose of SARS-CoV-2 vaccination.

Patient information is obtained from the NHS spine and undergoes validation to improve accuracy and completeness.

Death registration data for England and Wales are collated by the Office for National Statistics (ONS) (18). These data contain information relating to a death and the deceased individual. Causes of death, including underlying and primary causes, are provided as International Classification of Diseases (ICD-10) codes, based on the text provided on the death certificate (19).

Hospital Episode Statistics contains information on all NHS hospital admissions, Accident and Emergency (A&E) attendances, and outpatient visits in England (20–23).

Standardised coding of procedures, diagnoses, and medical conditions are captured for each episode, including information on primary diagnosis, treatment, comorbidities, and complications.

Supplementary Table 1 describes the features of all the datasets used.

### 2.2 Cohort building methods

To create a cohort of individuals who tested positive and negative for SARS-CoV-2, we apply entity resolution methods to identify unique individuals within the COVID-19 testing datasets.

These methods involve SGSS, USD, the NIMS denominator, and death registration data. They utilise PII and demographic information to create a dataset containing key identifiers for linkage to other data sources. The NIMS denominator acts as the gold standard for PII as information is obtained from the NHS spine, and death registration data provide validated PII for deceased individuals, capturing those who died prior to the creation of the national vaccine register. COVID-19 tests from March 2020 to April 2022, death registration records from March 2020 to January 2021, and NIMS records for those eligible for vaccination from December 2020 to April 2022 are included in the entity resolution process.

To maximise uniformity, datasets are initially cleaned, removing all non-alphanumeric characters and additional spaces present in name, NHS number, and postcode variables, and NHS numbers and postcodes are validated (24). The entity resolution process first runs between SGSS, USD, and NIMS and then, for records that have not been linked, between SGSS, USD, and death registration data. Each stage comprises five sequential iterations (Table 1) and nine sub-iterations (Table 2). Once linked, individuals are excluded from the following iterations. Each iteration includes deterministic matching of at least three person identifiers (of NHS number, sex, residential postcode, and date of birth) and deterministic matching and probabilistic matching (using the LIKE and DIFF SQL functions) of forename and surname.

**Table 1:** Description of entity resolution iterations 1.1 to 5.9 used in ECHOES cohort creation.

Entity resolution iteration	Person identifiers included in the linkage	Excluded/NULL fields
1.1–1.9	NHS number, forename, surname, dob, sex, postcode	
2.1–2.9	NHS number, forename, surname, dob, sex	Postcode
3.1–3.9	forename, surname, dob, sex, postcode	NHS number
4.1–4.9	NHS number, forename, surname, dob, postcode	Sex
5.1–5.9	NHS number, forename, surname, dob, sex, postcode	(spaces in the forename and surname fields removed)

**Table 2:** Description of entity resolution sub-iterations 1 to 9 used in ECHOES cohort creation.

	Person identifiers deterministically linked	Person identifiers probabilistically linked
1	NHS number, forename, surname, dob, sex, postcode	
2	NHS number, surname, dob, sex, postcode	forename (LIKE^*^)
3	NHS number, surname, dob, sex, postcode	forename (DIFF^**^)
4	NHS number, forename, dob, sex, postcode	surname (LIKE)
5	NHS number, forename, dob, sex, postcode	surname (DIFF)
6	NHS number, dob, sex, postcode	forename (LIKE), surname (LIKE)
7	NHS number, dob, sex, postcode	forename (DIFF), surname (LIKE)
8	NHS number, dob, sex, postcode	forename (LIKE), surname (DIFF)
9	NHS number, dob, sex, postcode	forename (DIFF), surname (DIFF)

* The LIKE function links two records if a specific character string (such as “Chris”) matches a specified character pattern (such as “Christopher”) (27).

** The DIFF function uses SOUNDEX to compare the difference between two character expressions (28). SOUNDEX converts string variables to four-character codes based on how the string sounds when spoken in English and can then be compared to other strings in terms of how they sound when spoken (29). For example, “Chris” would be linked to “Kris”.

The first iteration includes all identifiers (NHS number, forename, surname, DOB, sex, and postcode) to ensure the prioritisation of exact matching of records. Subsequent iterations exclude postcode, NHS number, and sex and consider alternative formatting of person identifiers. This accounts for changes in residence over the time period, different formatting of names across datasets (e.g., Anne-Marie vs. Anne Marie), and missing and erroneous fields.

The entity resolution process was developed iteratively, assessing the results of each linkage, including mismatches and unmatched records. Corrections were made, and subsequent iterations were developed to maximise the linkage of unmatched records. Where conflicts between what is recorded in datasets occur, PII from NIMS or death registration data is prioritised over testing data.

Following entity resolution, all PII (except the NHS number required for further data linkage) are removed from the dataset.

### 2.3 Censoring and exclusion criteria

Exclusion criteria and censoring rules are then applied. All records require a valid NHS number (verified against or obtained from NIMS or death registration data). Records without a valid NHS number were excluded from testing.

**Figure 1:**
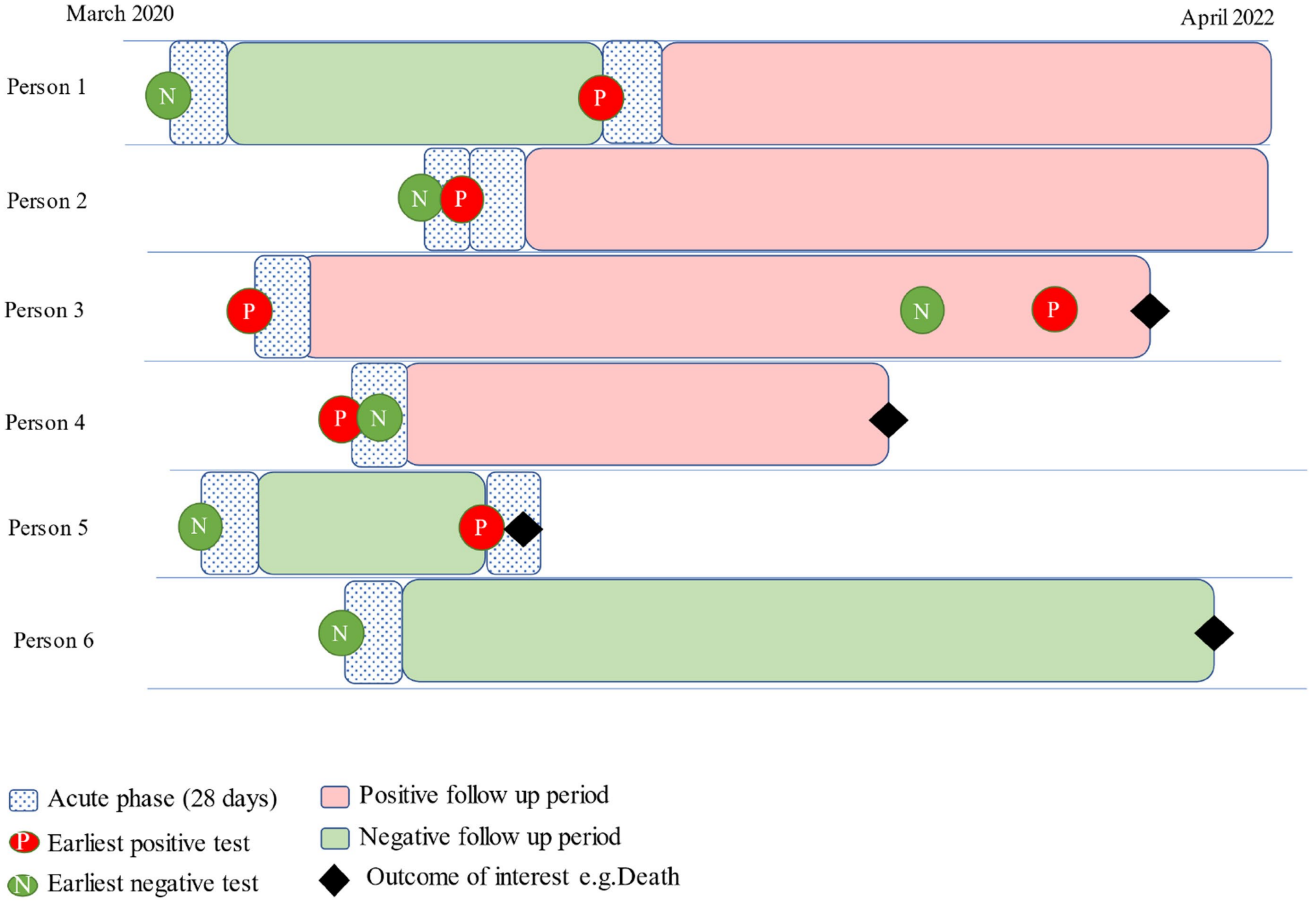
Example follow-up time sequence following positive and negative tests.

To investigate post-acute outcomes, individuals must have survived beyond the acute phase of infection, with follow-up starting 28 days after individuals’ earliest positive and negative tests. Data are at individual and test result levels, as individuals can contribute both positive and negative follow-up time. If an individual tests negative before their first positive, both tests are included (Person 1 in Figure 1). However, if the positive test is within 28 days of the negative test, the negative test is negated, and the positive test is retained (Person 2 in Figure 1). If an individual tests positive before they test negative, the negative test is ignored, and they are only followed up after their positive test (Persons 3 and 4 in Figure 1).

Follow-up time for each individual is calculated. For negatives, this is calculated from 28 days after their negative test, and whichever comes first of, (a) date of death, (b) a positive test or, (c) the health outcome of interest occurs. For positives, this is calculated between the earliest test date and the date of death or outcome occurrence (Figure 1).

### 2.4 Linkage to other data sources

The ECHOES cohort is linked (using NHS number) to hospital attendance data and death registration data to obtain information on a range of health outcomes and factors associated with SARS-CoV-2 infection. These include cause of death, pre-existing health conditions, comorbidities, and acute phase hospitalisation (Supplementary Table 1).

Death registration data provides the date and cause of death. Time to death is calculated, and cause of death ICD-10 codes are categorised into disease groups (25).

Vaccination status at the time of test is obtained from NIMS and can be defined as: i) fully vaccinated (having two or more vaccine doses) or unvaccinated, or ii) by time since the last vaccine dose received.

Linking the ECHOES cohort to HES allows a range of health indicators to be identified, including acute disease characteristics, pre-existing health conditions, and post-acute health outcomes. Hospital admission information at the time of the test can be used to indicate hospitalisation during the acute phase of COVID-19 and to determine whether testing was carried out in a hospital setting. Diagnoses ICD-10 codes recorded in HES prior to an individual’s test can be used to indicate pre-existing conditions and comorbidities. Pre-existing conditions can be defined using the standard ICD-10 chapter categorisations or through clinical phenotyping, and comorbidity scores can be assigned using tools such as the Charlson Comorbidity Index (26). Furthermore, information on hospital admissions and procedures during the post-acute phase can indicate healthcare usage, and by applying disease area and condition categorisation methods, diagnosis codes during this phase can be used to define post-acute health outcomes at different time points after an individual’s initial SARS-CoV-2 test.

### 2.5 Analytical method

The aim of analyses using these data is to compare the risk of post-acute health outcomes between those who tested positive and negative for SARS-CoV-2.

Using this cohort, a range of study designs can be used to assess different outcomes within an individual’s follow-up time. A matched cohort design can be applied, matching individuals who test positive and negative on a range of characteristics, depending on the objectives of the study.

## 3 Results

**Figure 2:**
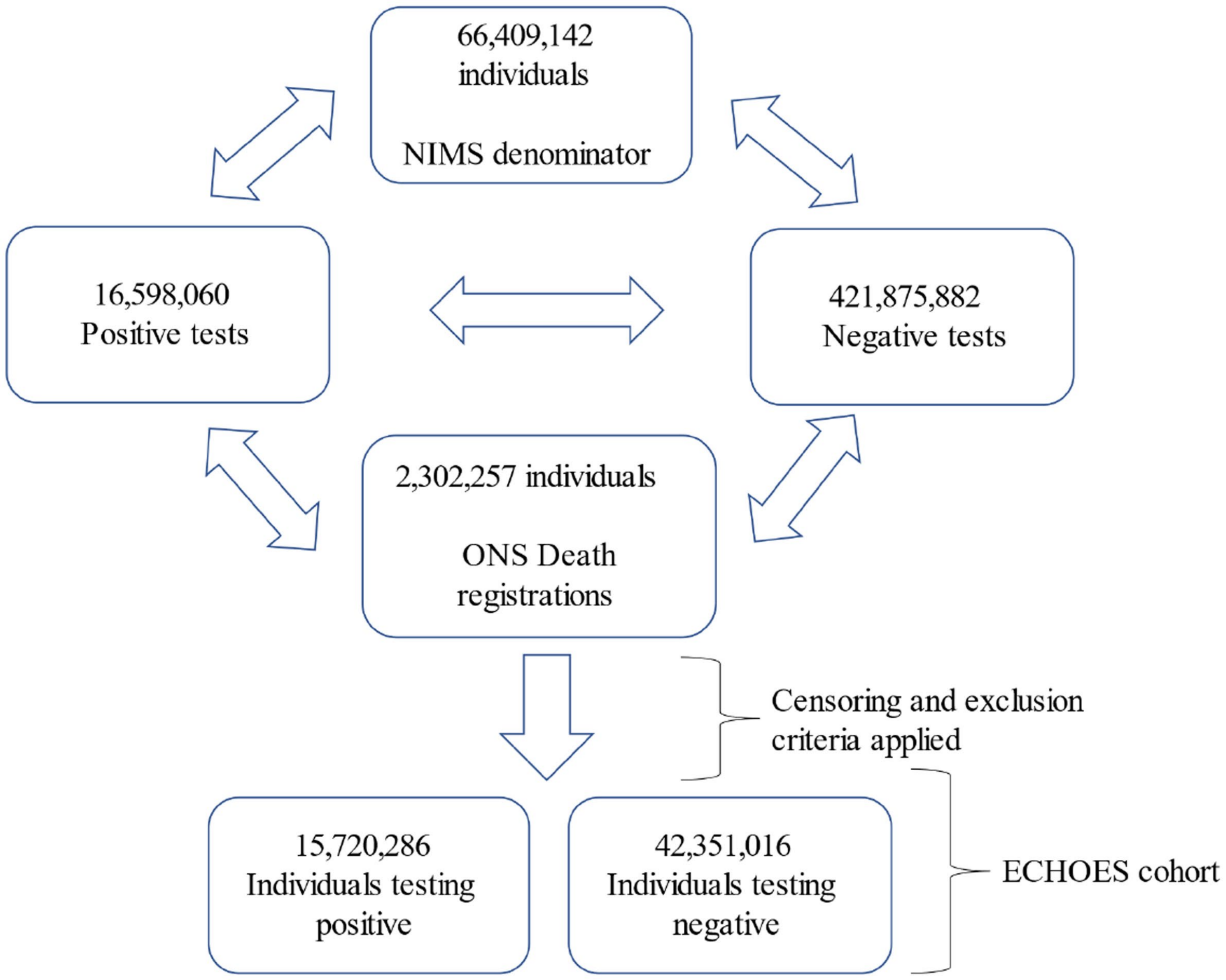
Flow diagram of test records included in the entity resolution phase and the resulting ECHOES cohort.

The entity resolution process using 16,598,060 positive episodes and 421,875,882 negative tests recorded at the time of dataset development resulted in 92.8% of positive records and 85.7% of negative records who tested between March 2020 and April 2022 being linked to a person record. Following censoring and exclusion, 44,234,762 unique individuals tested for COVID-19 are included. As individuals can be included in the positive and negative groups, this represents 15,720,286 positive individuals and 42,351,016 negative individuals (Figure 2). When assessing follow-up to 27 September 2023, the median follow-up time was 665 days (IQR: 619–806) amongst those testing positive and 985 days (IQR: 449–985) in those testing negative.

Within this same follow-up time, 136,814 (0.88%) positives and 156,376 (0.40%) negatives died in the acute phase of COVID-19 infection (within 28 days of their earliest test). These individuals will be excluded from analyses. 249,683 (1.60%) positives and 914,237 (2.35%) negatives died within the post-acute phase (more than 28 days of COVID-19 test) within the follow-up period.

### 3.1 Characteristics of the ECHOES cohort

**Figure 3:**
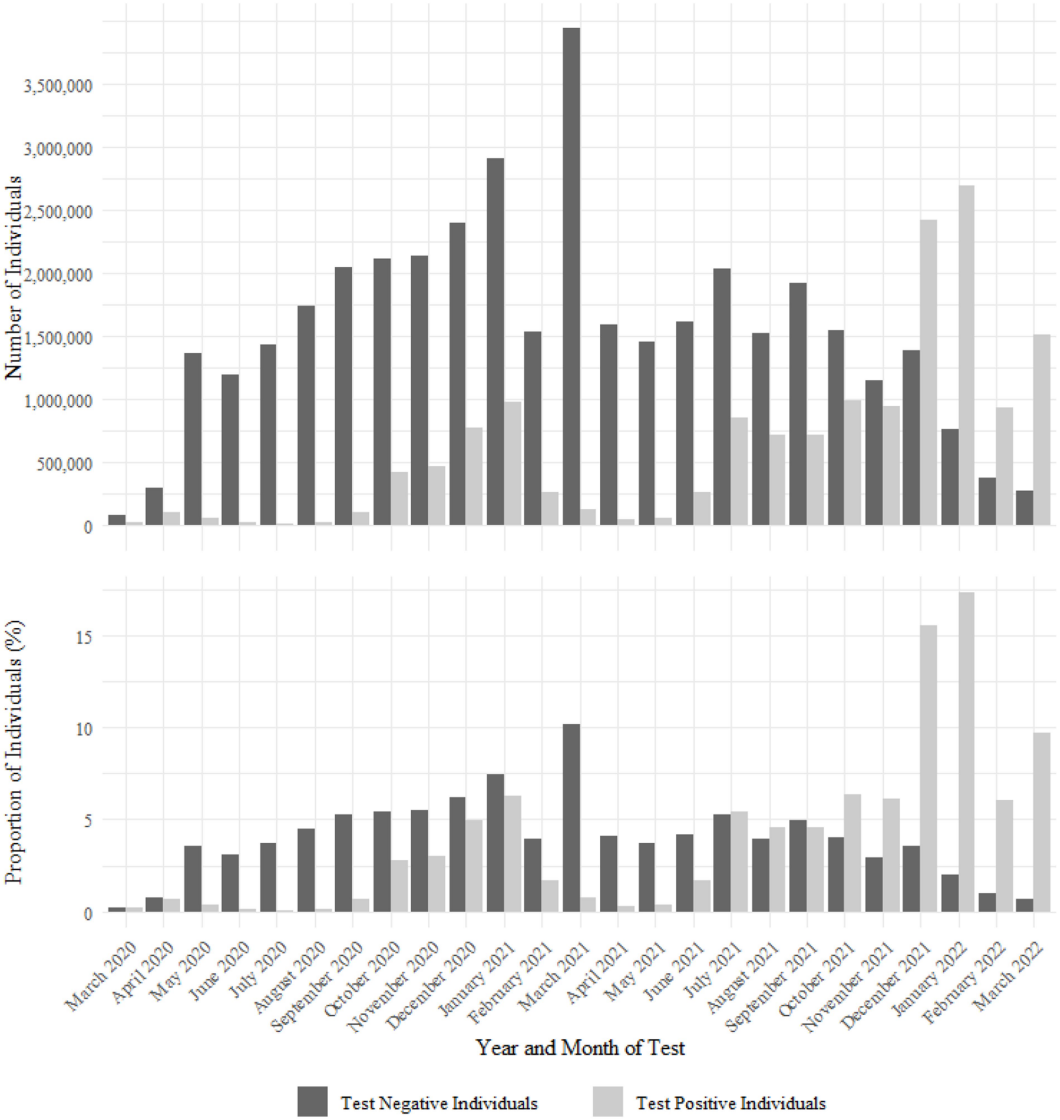
Number and proportion of positive and negative SARS-CoV-2 tests in the ECHOES cohort by earliest test date.

The number of individuals testing negative was highest at the start of the study period, between March 2020 and March 2021, and proportionally, individuals testing negative in the cohort were skewed towards the earlier part of the pandemic (Figure 3). The number of individuals testing negative each month was consistently higher for most of the pandemic period. The number and proportion of individuals testing positive were much higher from December 2021 onwards, coinciding with the emergence of the SARS-CoV-2 Omicron genomic variant (Figure 3).

The ECHOES cohort contains slightly more females than males, with 53.7% of the test-positive individuals and 52.3% of the test-negative individuals recorded as female.

The median age of those tested positive and negative was 35 (IQR: 19–50) and 39 (IQR: 21–57), respectively.

At their earliest test, 22% of test-positive individuals compared to 20% of test-negative individuals were aged under 18 years. Similarly, 27% of test-positive individuals and 24% of test-negative were aged 18 to 34 years; 37% of test-positive and 34% of test-negative were aged 35–59 years, and 11% of test-positives and 18% of test-negatives were aged 60–79 years; 2.7% of test-positive and 4.8% of test-negative individuals were aged 80 years and over.

The highest proportion of the cohort were residents in the Midlands, with 18.7% of test-positive individuals and 18.2% of test-negative individuals, followed by 15.8 and 16.6% of residents in the South East of England, respectively. The smallest proportion of the cohort were residents in the South West, with 9.5% of positives and 10.3% of negatives. Both positive and negative groups were fairly evenly distributed across IMD decile groups.

At the point of the earliest test, 46% of positives and 20.8% of negatives were fully vaccinated.

A breakdown of the demographic characteristics of the ECHOES cohort is shown in Supplementary Table 2.

## 4 Discussion

The development of the ECHOES dataset facilitates the investigation of a range of post-acute health outcomes, including pre-specified health conditions and mortality. This will allow a greater understanding of how a SARS-CoV-2 infection is associated with changes in the risk of health outcomes, potential determinants for these outcomes, and the long-term impacts of COVID-19 more generally. Analytical plans using the ECHOES dataset involve first assessing the risk of death in the post-acute phase at different time intervals using a matched cohort design, matching on a range of demographic factors. Then, further assessing specific population groups most at risk of post-acute death and the risk factors associated. These methods will then be applied to assess diagnosed clinical conditions captured in secondary care, including broad disease groups such as neoplasms and circulatory, respiratory, and nervous system diseases.

The inclusion of national-level COVID-19 testing data in the ECHOES dataset creates a representative dataset, mirroring the overall population in England. Furthermore, the ECHOES dataset captures both those testing with asymptomatic infection and those with more severe disease testing in hospital settings.

A major strength of ECHOES is its large size. This facilitates the investigation of rare outcomes and outcomes amongst specific population groups. Using person-level data from multiple data sources allows comprehensive linkage methods to be developed to create an accurate testing record for each individual and allows a robust control group to be created. This adds validity to the results produced and allows multiple study designs to be applied.

Additionally, multiple data sources allow various health outcomes and risk factors to be investigated. Demographic factors such as age, sex, residential location, ethnicity, and IMD can be considered, alongside vaccination status, comorbidities, pre-existing health conditions, acute disease severity, and the SARS-CoV-2 variant. Moreover, the outcomes identified will not have to be considered independently and can be considered indicators of future health outcomes.

Despite capturing all COVID-19 tests in England, only those with a valid NHS number are included. This may exclude specific population groups, such as those not engaged in healthcare. However, as ECHOES aims to compare outcomes between positive and negative groups rather than providing robust prevalence estimates for outcomes, this is unlikely to significantly impact the results produced. Stratifying analyses by demographic characteristics will help us to understand the impact of this limitation on the results produced.

As the ECHOES dataset is based on positive and negative SARS-CoV-2 testing data, the impact of under-reporting both positive and negative SARS-CoV-2 tests needs to be considered when interpreting the results.

Certain population groups may have been more likely to not self-report their home test results. This could be related to entitlement to sick pay and other barriers to testing.

Furthermore, early in the pandemic, before the rollout of self-administered tests, testing was limited to PCR tests in healthcare settings (March to June 2020), and non-hospitalised COVID-19 cases had limited access to testing. This may introduce inaccuracies in the results; however, these can be overcome by stratifying analysis by different time periods.

Changes in test availability may also lead to underrepresentation of population groups and misclassification of individuals. For example, individual’s earliest positive tests captured in ECHOES could be reinfection, and individuals who tested negative may have a prior unreported positive test.

Finally, the dataset is designed for long-term follow-up of individuals, with periodic linkage to updated health outcome data. As widescale community testing was ceased in April 2022 in England but COVID-19 has continued to circulate in the population, individuals infected with SARS-CoV-2 after this date may not be captured, resulting in potential misclassification of the negative group over time and a reduction in risk difference between the two testing groups for certain outcomes.

Despite these limitations, the ECHOES dataset offers a robust and comprehensive tool for investigating COVID-19 post-acute health outcomes in England. The dataset’s strengths will significantly enhance our understanding of the long-term impacts of SARS-CoV-2 infection.

## Data Availability

Any research conducted using the ECHOES dataset must be done as a formal academic collaboration between UKHSA and an academic institution. Proposals for collaborations will be considered by the COVID-19 Epidemiology team to evaluate the scientific quality and feasibility of the proposal.
